# Key Features of Successful Research‐Related Roles for Nurses and Midwives in out of Hospital Settings: A Mixed Methods Approach

**DOI:** 10.1111/jan.70021

**Published:** 2025-07-01

**Authors:** Louise Wolstenholme, Mary Alvarez, Ruth Endacott, Declan Robinson, Catherine Henshall

**Affiliations:** ^1^ National Institute of Health & Care Research, Nursing & Midwifery London UK; ^2^ Sheffield Children's NHS Foundation Trust Sheffield UK; ^3^ North Bristol NHS Trust The Chilterns, Southmead Hospital Bristol UK; ^4^ Oxford Institute for Applied Health Research Oxford Brookes University Oxford UK

**Keywords:** community, midwives, nurses, research capacity building, settings, the research capacity development for impact framework

## Abstract

**Aim:**

To explore the views of health care professionals involved in initiatives that have led to successful research‐related roles for nurses and midwives working in community settings.

**Design:**

A sequential mixed‐methods study.

**Methods:**

Between December 2022 and January 2023 a survey was completed by health care professionals with relevant experience of successful research‐related initiatives for nurses and midwives in community settings. Survey responses were categorised into low, medium or high‐priority examples of productive practice. Nineteen of the twenty responders who provided high‐priority examples were interviewed between May and July 2023. The research capacity development for impact framework underpinned data collection and analysis. Data were thematically analysed using the framework method.

**Results:**

The seven themes of the research capacity development for impact framework: leadership and sustainability, skills and confidence building, infrastructures, linkages and collaborations, ownership and responsibilities, actionable dissemination and co‐production were identified as important features of successful research‐related roles in community settings. A new cross‐cutting theme of trust and relationships was generated. The initiation, continued growth and ambition continuum guided the development of the planning change and features of success template.

**Conclusion:**

This study highlighted the key features that matter when planning change and developing research‐related roles for nurses and midwives in community settings.

**Impact:**

Study findings have the potential to inform policy and practice for organisations focused on developing research capacity and capability in community settings.

**Reporting Method:**

This study adhered to the COREQ reporting guidelines.

**Patient and Public Involvement:**

No patient or public involvement.

**Summary**

- What problem did the study address?
  ○ Research visibility, activity and delivery of studies is limited within community settings.
  ○ There is an increasing ambition to focus on ‘out of hospital’ settings for research, and a co‐existing need to understand how best to build research capacity and capability in these settings to realise this potential.
- What were the main findings?
  ○ Findings from the initiatives highlighted that most organisations were still in the initiation phase of building research capacity and capability in community settings.
  ○ Whilst success is attributed to the initiatives involved in the study, most acknowledged the need for ongoing and greater investment in research for growth to be sustained and ambitions achieved.
- Where and on whom will the research have an impact?
  ○ Study findings and the ‘planning change and features for success’ template can help guide and support clinical and research leaders in community settings, to build nurses and midwives research capacity and capability.
- What does this paper contribute to the wider global clinical community?
  ○ Improving care and health and well‐being outcomes through research undertaken in ‘real world’ settings such as homes, care homes, schools is crucial locally, nationally and internationally.
  ○ Raising research visibility and identifying the features that matter to build research capacity and capability for nurses and midwives in community settings has global application and relevance.

## 1 Introduction

Nursing focused research provides the scientific foundation for evidence‐based nursing. It informs clinical decision‐making, ensuring that care is based on the best available evidence. Boaz et al. (2015) suggested that having research as part of organisational structures matters for healthcare performance and describes this as a ‘by‐product’ of the research itself. The National Institute of Health and Care Research (NIHR) clearly states the ways in which research can help address health and social care challenges, whilst recognising that this research needs to be practically and meaningfully embedded within health and social care services (NIHR 2021). In recent years there has been a greater emphasis on research not solely being carried out in hospitals but instead being carried out within ‘real‐world’ settings such as homes, schools, care homes and hospices, as a means of reaching underserved regions and communities (NIHR 2021). Community nursing research is a growing area of international interest, with the International Collaboration for Community Health Nursing Research (ICCHNR 2023) asserting the importance of increasing the visibility of nursing research in community settings.

## 2 Background

In November 2021, the Chief Nursing Officer for England launched their inaugural strategic research plan ‘making research matter’ (NHS England and NHS Improvement 2021). At the centre of the strategic plan is the ambition to create a people centred research environment that is supported by nurses with the skills to participate in, deliver and lead research. This was complemented in 2023 by the Chief Midwifery Officer for England's Strategic plan, which set out to embed maternity and perinatal research in clinical practice (NHS England 2023). Other national policy documents, such as ‘best research for best health: the next chapter’ (NIHR 2021) identified the need to enhance and develop the research capacities and capabilities of underrepresented staff groups working in NHS and non‐NHS settings, with particular emphasis on community, public health, primary care and social care. To enact some of these key policies, core programmes of work are required to promote and provide the right sort of research infrastructure to support nurses and midwives to be research active within a range of health and social care settings. This research activity can take multiple forms at all stages of nursing and midwifery careers, encompassing a breadth of initiatives, all of which can contribute to changing the research culture across the nursing and midwifery professions (NHS England 2022).

Strengthening and supporting the research capacities and capabilities of nurses and midwives by facilitating better research infrastructure in out of hospital settings is crucial for accelerating the generation of new evidence, as well as for addressing health challenges and inequalities within local communities (NIHR 2021). Research undertaken in community, public health, primary care, and social care settings requires viewing through a different lens from research undertaken in secondary care. In out of hospital settings research is less visible and opportunities for, and commitment to research engagement, activities and roles are less evident (Bowers 2018; Henshall et al. 2022; Hoverd et al. 2023). The evaluation report (Menzies et al. 2022) from the NIHR's Senior Nurse & Midwife Research Leader Programme (70@70) highlighted the need for more research activity to be developed, implemented and sustained across out of hospital settings, by developing more nurse and midwife research leaders in these settings. This led to the development of the James Lind Alliance Priority Setting Partnership in Community Nursing (Henshall et al. 2022), which identified key priority topic areas of focus within the community from the perspectives of community nurses, patients and carers. However, despite the identification of these priority areas, a greater understanding of the various barriers and enablers to operationalising research activity within community settings is required.

Research‐related roles can encompass a variety of initiatives or activities that have some level of research involvement. Examples include nurses and midwives who undertake hybrid working between clinical and research, research champion programmes, research leadership roles, research capacity building roles and staff engagement with research networks. Communities of Practice can provide a forum through which research activities and engagement can occur and they support active research involvement (Hoverd et al. 2023). Similarly, research networks can support ‘close to practice’ research in four ways through: research delivery, research use in practice, developing and leading research and co‐production and priority setting (Wolstenholme et al. 2022). This can have widespread benefits, including increasing recruitment to clinical trials, with a study by Alvarez et al. (2023) finding that pregnant women were more likely to participate in research if they were offered research opportunities by community midwives with whom they had trusting relationships. However, despite the clear need for more research to be undertaken by nurses and midwives in out of hospital settings, it is unclear what key features of successful research‐related roles look like. This was the focus of the study reported on in this paper.

## 3 The Study

### 3.1 Aim

The study aim was to explore the views of health care professionals (HCPs) to identify the key features contributing to successful research‐related roles for nurses and midwives working in community settings.

Throughout the study the term ‘community settings’ relates to and is inclusive of all community, public health, primary care and social care settings and can also be referred to as ‘out of hospital’. Nurses and midwives undertaking research‐related roles in these settings do not neatly fit into similar categories; instead, there are a wide variety of employers and host organisations with varying degrees of research infrastructure. This can lead to challenges in identifying and accessing the necessary information. Therefore, this study took an inclusive and encouraging approach to identifying, highlighting and learning from areas where successful research‐related roles for nurses and midwives in those settings were happening.

### 3.2 Research Question

What are the key features of successful research‐related roles for nurses and midwives working in community, public health, primary care and social care settings?

## 4 Methods/Methodology

### 4.1 Study Design, Setting and Population

The study undertook a sequential mixed‐methods approach consisting of a survey and interviews, utilising an exploratory design, incorporating thematic analysis and the framework method (Gale et al. 2013). The purpose of the survey was to gather representative data about the breadth and variety of initiatives being undertaken across England and to identify relevant participants who could provide further information at interview. Initiatives identified as high‐priority examples, and invited to interview, provided the in‐depth descriptive data that was analysed to identify the key themes that matter for successful research‐related roles for nurses and midwives in community settings.

### 4.2 Rigour

To guide the study process, the RISE (**R**esearch **I**n Community **SE**ttings) national steering group was established, led by a NIHR Nursing & Midwifery Clinical Fellow and lead researcher (L.W.). The group comprised a range of national stakeholders who were well placed to understand the context of the topic area, recognised the challenges of identifying and accessing potential initiatives, were immersed in the field enabling them to have informed views, and were able to reach out to other potential contributors. Survey and interview data analysis was led by the lead researcher (L.W.), in collaboration with the wider research team, to ensure a range of views and perspectives informed data interpretation.

### 4.3 Theoretical Framework

**FIGURE 1:**
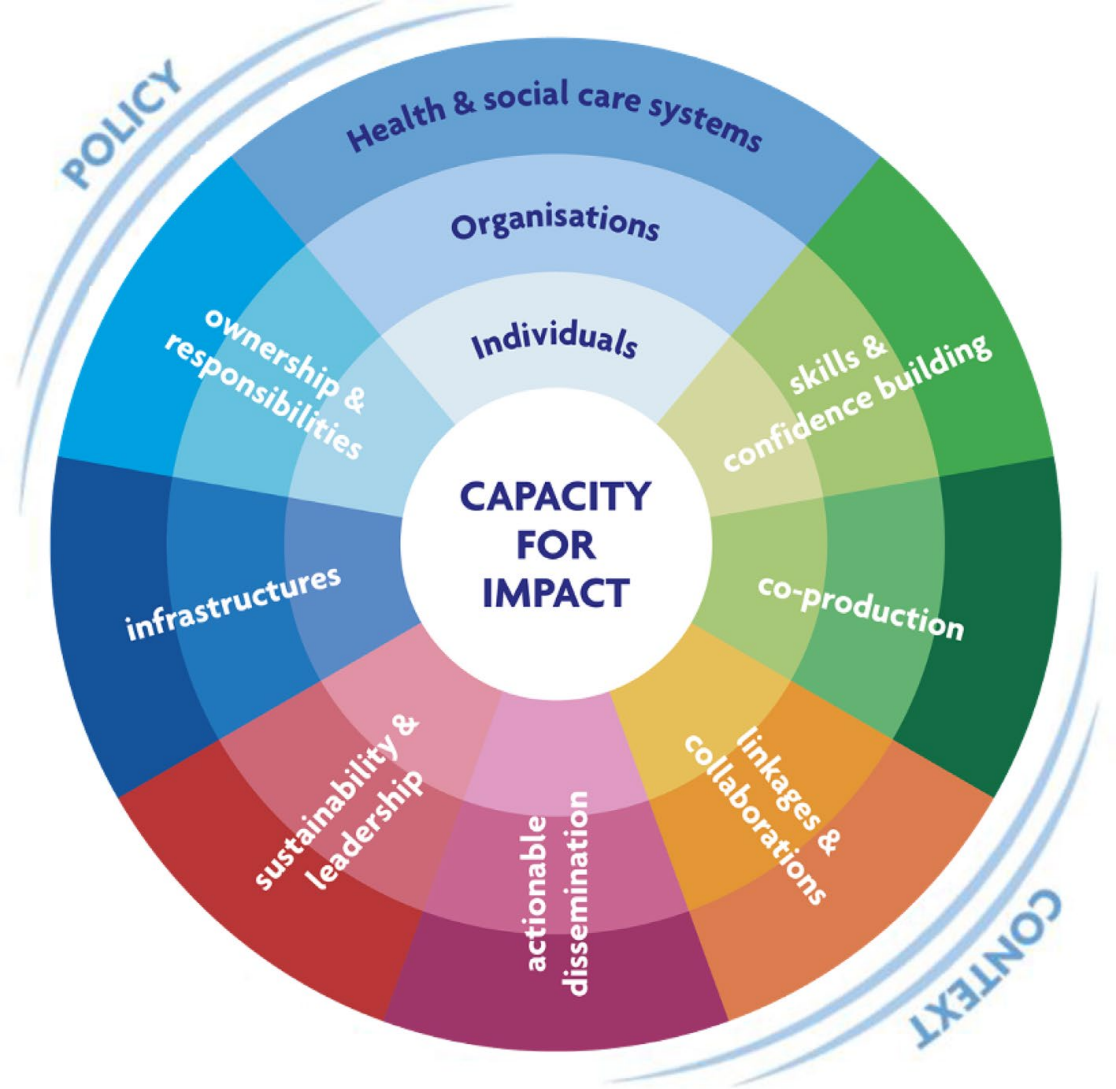
A framework for RCDi. RCDi, research capacity development for impact.

Various theoretical models and concepts have been developed in recent years to support the development of research capability and capacity building initiatives for nurses and midwives at a national and international level (Cooke 2005; Lode et al. 2015; Chen et al. 2019; Whitehouse et al. 2022). One such theoretical model developed over two stages is Cooke's work on research capacity building (2005, 2021). Cooke's initial model (2005) identified six principles of research capacity building and was generated through analysis of policy, literature, empirical studies, and discussions with a research support unit. ‘Re‐imagining’ of the framework led to the development of the *RCDi: research capacity development for impact* framework (Cooke 2021), with a focus on seven principles that provide opportunities for planning change, building research capacity and measuring service impact (Figure 1). In this study, the seven principles underpinned the development of the interview topic guide, as well as providing the theoretical structure against which the data analysis process was mapped.

### 4.4 Ethical Considerations

An NIHR governance officer approved the study, which satisfied the governance procedures required by the NIHR as the sponsor organisation. The online survey was produced in line with General Data Protection Regulation (GDPR) and was written in plain English. Survey completion by responders implied consent. Prior to the interview stage, potential participants, who had given permission to be contacted during the survey stage, were sent a participant information sheet and consent form to complete and return via email, before being invited to interview. The project presented no major ethical dilemmas, and ethical principles in research were adhered to in relation to informed consent, integrity, confidentiality and data protection, conflicts of interest and respect for persons.

### 4.5 Stage 1: Survey

#### 4.5.1 Survey Development

Developing the RISE survey, available in Appendix S1 in full, was an iterative process, involving discussions with the RISE national steering group. Steering Group discussions informed survey development regarding content format and use of terminology. Specific feedback emphasised the need to include tailored and inclusive language as a means of engaging survey recipients. It was agreed that a mixture of quantitative and qualitative data capture fields was required to gather the necessary breadth and depth of data; this included information on who the initiative was for, its purpose, aims, anticipated outcomes, funding sources and outputs. It was also agreed that in terms of demographic data collection, the survey should collate data relating to specific initiatives identified rather than the focus being on the survey responder. Hence, limited information was asked about the responder, but data was collected regarding their role and the setting they worked in. To guide respondents and aid completion, the survey included relevant examples such as: a district nurse undertaking a research capacity building role, or a research midwife having a focus on delivering community‐based research.

#### 4.5.2 Access, Recruitment and Data Collection

The eligibility criteria for survey responders was broad. Responders needed to be an HCP who had been involved in a research initiative within a community setting with a focus on research engagement for nurses and midwives. The RISE online survey was open for six weeks, from December 2022–January 2023, and completion was via a Google Form disseminated through several routes. One route was via the ‘We are NIHR @NIHRcommunity’ on Twitter (prior to name change to X). Further routes included the national Steering Group's professional networks; all stakeholders identified and committed to disseminating the survey (e.g., CHART—Community Healthcare Alliance of Research Trusts, QNI—Queens Nursing Institute, NHS England Matrons group). A final route was recipients were encouraged to share the survey across their organisations and networks, as a means of snowball sampling. A reminder was sent through Twitter one week before the survey was due to close. Survey completion and submission implied consent to participate as outlined in the content of the survey.

### 4.6 Data Analysis

The lead researcher (L.W.), experienced in qualitative research methods, extracted the free text survey data, analysed it descriptively and tabulated it in Microsoft Excel according to the nurse/midwife group that the initiative was for (e.g., community nurses, mental health nurses, community midwife, social care nurse) and organisational setting (e.g., Community Trust, Mental Health Trust), alongside a brief description of the initiative. This tabulated data was shared with the RISE steering group who, to enable identification of potential participants for interview, had devised a ‘productive practice’ definition. Productive practice was determined as a role, initiative or activity that demonstrated a link to research, involved nurses and midwives based in or working in a community setting and provided the potential for new learning in relation to creating successful research‐related roles in community settings. The example could demonstrate all the above (high priority), majority of the above (medium priority) and some of the above but with limitations (low priority). Initially the productive practice definition was applied to six survey responses by the RISE steering group members during a meeting, to ensure consensus before the research team (L.W. and M.A.) categorised the remaining surveys. Applying the productive practice definition to the tabulated data of the initiative, which included purpose, aims, role remits, achievements, funding sources etc., enabled each initiative to be categorised into high, medium or low examples.

### 4.7 Findings

A total of 64 survey responses were received, 62 through completion of the Google Form in response to the original tweet and two via email through snowball sampling techniques. Table 1 indicates the organisational setting of the initiatives identified, the areas of work/roles of survey responders and the nurse/midwife groups that the initiative related to. The quantitative survey findings from the research engaged initiatives informed the qualitative stage of the study by helping to identity the key research initiatives that required further in‐depth exploration. Of the 64 survey responses, 34 (53%) responders provided examples of initiatives that detailed key features of successful research‐related roles for nurses and midwives working in community settings. Of these, twenty provided sufficient detail to be categorised as examples of high productive practice (Table 2a). The remaining 14 examples provided information that demonstrated relevance but were categorised lower due to the information provided lacking detail, being aimed across a broader range of healthcare professionals, having a limited community focus, or a specific topic focus. Thirty responses were categorised as non‐relevant examples (Table 2b).

**TABLE 1:** Organisational setting and areas of work/role of survey respondent.

		Number of responders' *n* (%)
Organisational setting of the role, initiative or activity	Acute	4 (6)
	Acute & community	15 (23)
	Care home	2 (3)
	Clinical research network	5 (8)
	Community	12 (19)
	Community & mental health	6 (9)
	Community, mental health & learning disabilities	8 (12)
	Community/university	1 (2)
	Community/local authority	1 (2)
	Integrated care board (ICB) & NHS England/NHS improvement	2 (3)
	Local authority/social enterprise	2 (3)
	Mental health & learning disabilities	1 (2)
	Primary care	2 (3)
	University	3 (5)
Total		^*^64 (100)
Area of work/role of survey responder	Academia	3 (5)
	Advanced clinical practitioner/research nurse	1 (2)
	Clinical educator	1 (2)
	Clinical fellow (research)	2 (3)
	Nurse or midwife/clinical academic	2 (3)
	Occupational therapist & psychologist	2 (3)
	Research champion (clinical)	3 (5)
	Research facilitator/training manager	3 (5)
	Research manager	9 (14)
	Research nurse/midwife/matron/clinical research practitioner	15 (23)
	Nurse/midwife/nurse specialist/nurse consultant/advanced clinical practitioner/nurse director	23 (35)
Total		^*^64 (100)
Nurse/midwife group identified in relation to role, initiative or activity	Community (district nursing)	17 (25)
	Community specialist nurse (e.g., tissue viability, respiratory)	3 (4)
	Community midwives	6 (9)
	0–19 Public health (health visitor and/or school nurses)	10 (15)
	Primary care nurses (practice nurses)	7 (10)
	Community paediatric nurses	6 (9)
	Community Sexual health nurses	3 (5)
	Community mental health nurses	9 (13)
	Social care nurses/care home nurses	5 (8)
	Learning disability nurses	1 (2)
Total		^*^67 (100)

*Note:* *n = 67 as some initiatives were aimed at more than one professional group.

**TABLE 2a:** Relevant examples of productive practice initiatives.

Productive practice category	Relevant examples of productive practice initiatives	Total *n* (%)
High	Research champion roles/programmes, research capacity and capability roles, community research nurse roles, mentorship programme, research link workers	20 (59)
Medium	Reflective reading club for nurses and AHPs, Advanced Nurse Practitioner role in General Practice, Agile research team, Acute & Community research delivery project, Principal Investigator role development across all health care professionals	8 (23)
Low	Staff research fund to develop projects, a critical appraisal group, student nurse placement	6 (18)
Productive Practice initiative examples overall		34 (100)

**TABLE 2b:** Non‐relevant examples of productive practice initiatives.

	Not relevant examples of productive practice initiatives	Total *n* (%)
No research involvement	Service improvement	8 (27)
	Peer support group	1 (3)
	Education focus	4 (13)
Individual study or project	One off project/research study	8 (27)
	PhD/Masters	7 (23)
Research delivery	Research delivery of studies	2 (7)
Non‐productive practice initiative example overall		30 (100)

### 4.8 Stage 2: Interviews

#### 4.8.1 Access and Recruitment

Stage 2 of the study involved the collection of qualitative data through semi‐structured interviews. The interview sample size was determined through Malterud et al. (2015) concept of ‘information power’ (IP). IP proposes that a lower number of participants is needed if the power of the information that the sample holds is higher. The RISE steering group reviewed the survey responses in relation to the five IP factors; aim, specificity, theory, dialogue and analysis and agreed that for pragmatic reasons and informed by the IP factors, only the responders assigned to the high‐priority category, productive practice examples, were invited to interview. Considering ‘information power’, it was anticipated that data saturation would be realised through the rich interview data collated from the high‐priority participants. However, should new themes have continued to emerge, respondents who had provided medium priority examples of productive practice would have been approached for interview. The participant information sheet (Appendix S2) and consent form (Appendix S3) were reviewed and agreed by the national RISE steering group prior to distribution and were sent via email to survey respondents who had agreed to further contact (*n* = 20). Following consent, an invitation to interview (Appendix S4) and a topic guide (Appendix S5) were sent to participants. An initial pilot interview was undertaken with one of the participants, and a further 18 participants consented, with 19 conducted in total.

### 4.9 Data Collection

Online, semi‐structured interviews via Microsoft Teams were undertaken over a three‐month period (May–July 2023). Interviews were recorded using the audio‐visual function on Microsoft Teams. They were conducted by the lead researcher (L.W.), on a one‐to‐one basis and lasted approximately one hour. Additional notes were made during and after the interview. The semi‐structured topic guide was framed around the seven principles of the RCDi framework (Cooke 2021) for example ‘How much did skills and confidence building matter for your nurses and/or midwives in taking part in your successful research initiative in a community setting? What did this look like?’. The purpose of the interviews was to gain greater understanding from participants (*n* = 19) of some of the key features that enabled successful research‐related initiatives for nurses and midwives in community settings. Demographic data from the initial Stage 1 survey was extracted for the high‐priority examples.

### 4.10 Data Analysis

Interviews were transcribed on Microsoft Teams. Transcriptions were revisited by the lead researcher (L.W.) to ensure the notes were verbatim for analysis purposes. This stage of transcribing was undertaken mainly by the lead researcher (L.W.) as a means to being fully immersed in the data, with some also being undertaken by the project administrator. During the analysis phase, qualitative interview data was mapped against the linked quantitative survey findings to check alignment across the two data sets and to add richness to the initial survey findings. The survey data was reviewed prior, during, and post interview data collection to ensure an iterative and integrative approach to data collection and analysis. The Framework Method (Gale et al. 2013) was utilised to analyse the data. The stages of transcription, familiarisation with the interview, coding, developing a working analytical framework, applying the analytical framework, charting data into the framework matrix and interpreting the data, were employed. An initial coding framework was created and the seven themes outlined were deductively identified from the seven principles of the RCDi framework (Cooke 2021). Data was collated on a Microsoft Excel spreadsheet. This process of analysis deductively framed the themes and subthemes around suggested criteria (Cooke 2005), whilst also allowing inductively derived themes and subthemes (Appendix S6) to be generated. Whilst the lead researcher (L.W.) was primarily responsible for coding the data, regular feedback sessions were held with a second member of the research team (CH). This collaborative coding process helped refine the analysis and ensured that key themes were accurately and comprehensively represented. The findings, in relation to the identified themes, were re‐framed against three distinct phases of development: initiation, continued growth and ambition, as part of a ‘planning change continuum’.

### 4.11 Findings

Demographics around the different organisations and types of initiatives across the various ‘out of hospital’ settings were collated for the interview participants from their initial survey response (Table 3). The following ‘a priori’ themes used to present the findings were the seven principles derived from the RCDi framework (Cooke 2021): leadership and sustainability, skills and confidence building, infrastructures, linkages and collaborations, ownership and responsibilities, actionable dissemination and co‐production. An additional theme of ‘trust and relationships’ emerged from the analysis, with findings that were distinct from the other themes. The process of framework analysis, creation of themes, subthemes and attributing examples of productive practice, identified variation in the quantity of data generated in relation to the seven themes, resulting in some being represented in the data more than others. The following findings are presented in order of the frequency of the number of extracts attributed to each theme. Each data example is identified: ‘*Int’* refers to interview number and after the/the area of focus of the initiative (e.g., Public Health).

**TABLE 3:** Different organisations and types of initiatives across the various ‘out of hospital’ settings.

Initiative area of focus		Community *n* (%)	Community midwifery *n* (%)	Public health *n* (%)	Primary care *n* (%)	Social care *n* (%)
Organisation based in	Acute & community trust (*n*)	3 (16)	1 (5)	3 (16)		
	Community & mental health trust (*n*)	1 (5)		1 (5)		
	Community, mental health & learning disabilities trust (*n*)	2 (11)				
	Clinical research network (*n*)		1 (5)		2 (11)	
	Community trust (*n*)	2 (11)		1 (5)		
	Care home (*n*)					1 (5)
	University/community trust	1 (5)				
Total		19 (100)				
Types of initiatives	Research nurse/community nurse (hybrid roles) (*n*)	3 (16)				
	Research champion role/Programme (*n*)	2 (11)	1 (5)	2 (11)		
	Community trust R&D development (*n*)	1 (5)				
	Research capacity building (*n*)	1 (5)		3 (16)		1 (5)
	Community Research Fellow (*n*)	1 (5)				
	Research Link roles (*n*)	1 (5)				
	Community research nurses and midwives (*n*)		1 (5)			
	Practice nurse roles/Mentorship programmes (*n*)				2 (11)	
Total		19 (100)				

#### 4.11.1 Theme 1: Sustainability and Leadership

All initiatives identified by participants highlighted the importance of senior leadership and their ambitions to create a research environment and enable a positive research culture. Senior stakeholders included Chief Nurses, Medical Directors, Executive and Non‐Executive Directors and Trust Board members. A number of participants discussed the relevance of research drivers such as policy documents, mandated directives and national and local research strategies, in effecting change. Most initiatives were led from the Research and Development (R&D) department or individual clinical practitioners and not from clinical services management. Participants recognised that care delivery was the priority for clinical managers and that for research involvement to happen, and permissions to be granted, effort was required. Examples of navigating complexities and acknowledging the limitations of what could be achieved to ensure the overall success of the initiatives were common:

Obviously working with the clinical lead was quite pivotal and quite key because that enabled certain, you know permissions to take place across the service that ensured that she could kind of champion the programme at quite a high level across the service and that people would listen and kind of get on board. (Int 5/Public Health)

Sustainability of the initiatives created significant discussions by participants. Half the participants described initiatives as not at risk because they were either new, had high level endorsement, had been incorporated into someone's role, had secure funding or had in‐built flexibility so they could be sustained. The other half of the participants described concerns about initiatives not continuing and cited permissions, support, competing demands, lack of understanding, systems, processes, and a depleted workforce as potential reasons for this, also funding:

We put a new bid in for more funding [for my role]. We were successful, so I'm safe until 2024 and after that, we're obviously, we'll just keep reapplying for funding to see if we are successful, but yeah, I'm not really sure what will happen if we are not. (Int 8/Public Health)

#### 4.11.2 Theme 2: Skills and Confidence Building

Skills and confidence building were universally identified by participants as important to the success of the research‐related initiatives. Evidence of research awareness raising, through the sharing of information, was seen as a necessary output and a fundamental step for either the individual, within a team, the organisation or across systems and was described by most as being important to build confidence. Research engagement demonstrated an activity that involved meaningful interactions with contributory elements from different parties such as mentoring, one‐to‐ones, workshops and/or participating in a community of research practice. Some participants highlighted the specific importance of developing research skills; this varied from accessing training, to being a Principal Investigator and from structured to light touch programmes:

I [CRN (Clinical Research Network) Research Nurse] was over there every week or every two weeks building up a rapport, so you're demystifying things and they [practice nurses] realise actually, they've got an awful lot of skills and they can do the aspects much better than me going in from the outside. (Int 1/Primary Care)

However, the importance of research development opportunities was only mentioned briefly by seven participants. One participant spoke about an idea for secondments to the R&D department for six months before returning to practice; others spoke about research internships (for e.g., NIHR ICA Internship programme) and individuals undertaking academic study.

#### 4.11.3 Theme 3: Infrastructures

The creation of roles and initiatives was seen as important in relation to infrastructure. The breadth of examples described by participants varied in terms of how the initiative was conceived, what it entailed, whether it was a formal or informal appointment and the funding requirements. Some initiatives were strategically driven at both national and regional levels whilst others were locally motivated:

[The practice nurse mentor scheme] came about from a strategic action plan that was devised at an away day … the intended outcome is to make the nurse working on the research study on a day‐to‐day basis, feel supported and have a named accessible relatable person that they can go to, help them with any queries and hopefully point them in the right direction. (Int 7/Primary Care)

In relation to infrastructure participants described factors such as study availability, funding, R&D department involvement, community infrastructure, virtual/digital working and establishing links as all being key to progress the initiatives. The availability of appropriate community research studies was raised by participants and alongside this the need to develop research capacity and capability in underrepresented professional groups to be able to deliver community‐based studies:

What are the studies that can be generated that are low hanging fruit? … are there simple studies that answer important questions, you know, and those questions are very much often quality of life, not wonderful medical interventions in the community. (Int 18/Community)

Funding was fundamental, with almost all participants commenting on this issue as a key driver. Funding varied from role creation to individuals being awarded project work, to bespoke funding packages to progress and develop. A minority of participants mentioned being given dedicated time within their existing roles to implement their initiatives; however, they noted that resources were scarce and insufficient. Whilst R&D departments were not universally involved in all the initiatives, nearly half shared examples of R&D departments supporting the initiatives with resources whether or not they had any funding. For community research, many of the participants described vast organisations, covering a large geographical footprint with reduced research visibility, community infrastructure being complex and travel being lengthy. Findings linked to the relevance of digital and virtual working were raised by a few participants. One enthused about the benefits that digital working had brought to their organisation and the advantage of being community‐based:

There's a geographical challenge and I think that's one of the things that's perhaps unique to community settings, isn't it? It's the geographical challenge. (Int 5/Public Health)

#### 4.11.4 Theme 4: Linkages and Collaborations

Organisational linkages and collaborations created the most discussion and were highlighted by all participants as important. These varied across the different initiatives but the majority occurred for cross working, learning purposes, study delivery and capacity building. For some initiatives, across networks and systems working, including links between Clinical Research Network (CRNs) and practice were highlighted; this again varied in relation to study delivery, learning purposes and capacity building. The importance of national speciality working groups, regional primary care groups, regional speciality networks, and national champion groups were cited by many as important. For some of the initiatives, ‘leading’ these groups was their purpose, but many others highlighted the importance of being able to link into these groups:

We've obviously come along to the 0‐19 Research Network days, networking and getting a broader understanding of the health visitor and school nurse kind of world. (Int 8/Public Health)

The need for researcher and academic links was emphasised by over half of participants, with others stating that this was an ambition for the future. From a research delivery aspect, linkages and collaborations with Chief Investigators were cited; others quoted the roles of universities and academics from a learning purposes perspective and in developing research studies:

We're looking at is bringing the system (NHS Trust) to the university and inviting professors from across the university to come in and talk about their research to see if we can start building that integrated approach across the university to go alongside the integrated approach in the health service. (Int 16/Community)

#### 4.11.5 Theme 5: Ownership and Responsibilities

Leadership support to enable research ownership and responsibility was commented on by over half the participants; for the majority that leadership came from R&D departments and not clinical services. Some provided examples around ‘ownership from initiation’ and of being developed by individuals from practice, citing a bottom‐up approach; in these instances, senior leadership was not the driving force. Some of the participants recognised luck as important to success. Tenacity and self‐directed learning was discussed by over half of the participants; for those who led their initiatives from practice and described a bottom‐up approach, all but one linked this to personal growth:

I became aware that actually, there were senior nurses in other organisations that were really driving research. It was part of their kind of role and their job. And I just didn't feel we had that in the Trust. I felt we had a few key people who it was almost like their own personal interest as opposed to somebody professionally being responsible. (Int 15/Community)

Over half the participants viewed their initiative as a top‐down approach, where senior (organisational or research) leadership employed methods to create buy‐in from those in less senior positions. Within this, the majority recognised that there was an ambition to enable ownership and responsibility for those involved in the initiative. Creating buy‐in was described by some as problematic.

I'm definitely at the buy‐in point and that is difficult, I think one of the things that would be important is to move towards a kind of ownership thing, that is having studies that really resonate and really are important to whatever team. (Int 2/Community)

Some acknowledged that top‐down approaches were a necessary part of the initiative's progress. Others recognised the infancy of where their initiative was at and that feelings of uncertainty about research ownership and responsibility were to be expected.

#### 4.11.6 Theme 6: Co‐Production

Co‐production and shared working where researchers, practitioners, members of the public and other relevant individuals collaboratively develop research was mentioned as an ambition for over half of the participants. A few of the initiatives gave an example of co‐production that had occurred.

In another piece of work that I'm doing, which is with care homes, it's a ‘creating care partnership’, that's literally all about co‐production … we are literally directly working with a care home group to develop studies that are born out of their questions. (Int 9/Community)

Equally a few cited that co‐production was ‘not an ambition’ within the initiative and it had not been considered. Over half, though, mentioned co‐production as being ‘important’ for the future; some could articulate what that might look like, whilst others were uncertain how to go about it.

For us co‐production means producing something collaboratively with people with lived experience of mental health problems or dementia, or a carers or something. And I don't think we have done that very well in terms of our programme. (Int 11/Community)

#### 4.11.7 Theme 7: Actionable Dissemination

Actionable dissemination was considered an important theme for the success of the initiatives, with all but five participants providing examples of it. Two participants stated that they valued what dissemination could offer and had an ambition to develop in this way. Profile raising and increasing the visibility of research activity were highlighted as important for many of the initiatives, and most participants could provide examples, spotlighting research activities and creating awareness, celebrating achievements, sharing information on research learning and opportunities, and encouraging connections. This happened through a variety of methods such as email distribution lists, invites to learning sessions, posters, Trust Communications, bulletins about research studies, recorded showcase events, research stalls and attending groups and forums.

I think research stories are really helpful and it sort of demystifies it, it shows I'm a community nurse and I did this and if I did it you can do it. (Int 14/Community)

Measurable research outputs were described by over half of the initiatives, this varied from numbers of places on champion programmes, numbers of conference presentations undertaken and papers published, volunteer roles commenced and newsletters written. Over half of participants commented about an ambition for further growth around dissemination and shared examples of how and where that might be.

#### 4.11.8 Theme 8: Trust and Relationships

Several participants drew attention to the importance of trust and personal relationships. The building of relationships and creating trust was commented on by R&D department led initiatives looking to involve and engage clinical services in research activities.

I think maybe it's taking longer for me to build the relationship with our (community) services, maybe if I was somebody who was already working within the organisation and I might already have those relationships built or understand those services, then things would be quicker and you'd hope to have that trust and things which is where I guess me working in the teams might help. (Int 2/Community)

Others noted the authentic voice that a research interested individual in clinical services could offer:

It's very easy for me to go out to a team meeting and talk to a team about research, but actually if you've got someone within that team who's advocating for research, they have much more credibility, don't they? And perhaps would be taken … not taken more seriously but perhaps people would be more inclined to support them because it's their colleague rather than just a stranger turning up and telling them that they could be doing these things. (Int 12/Community)

## 5 Discussion

**FIGURE 2:**
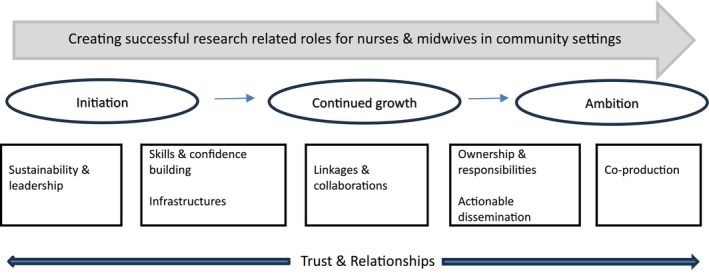
Initiation, continued growth, ambition continuum.

This study has demonstrated that for successful research‐related roles in community settings all seven principles of the RCDi framework (Cooke 2021) need to be considered. In addition, a new, cross‐cutting theme around trust and relationships is key to the success of these roles. The *initiation, continued growth, ambition continuum* (Figure 2) identifies three main phases. The participant interviews revealed that most resources, time and commitment were being expended during the *initiation* phase of the continuum, with a few initiatives describing some activity in *continued growth* or *ambition* phases.

Peckham et al. (2023) highlighted how a lack of organisational systematic approaches hindered research involvement and activity. Embedding evidence and research findings into practice is complex (Coates and Mickan 2020; Curtis et al. 2017; Greenhalgh 2018), where day‐to‐day care delivery is prioritised over the long‐term benefits to care that research can deliver on. Our findings, that research leadership for the initiatives detailed in this paper stemmed mainly from local R&D departments and individual's personal research ambitions, correlates with this perspective. A collective commitment to research activity from the community clinical teams was inconsistent and minimal; however, clinical leaders were shown to be key for permissions for research involvement, support and protected time. The benefits to inclusive leadership in empowering nurses to promote research capacity building is recognised (Henshall et al. 2022) and nationwide initiatives can increase research leadership within nursing and midwifery services (Henshall et al. 2020; Menzies et al. 2023). In relation to this, our findings showed that the interplay between organisational, clinical and research leadership requires careful navigation including acknowledging and being aware of workforce constraints, bargaining, being realistic and compromising, as part of a two‐way discussion between those leading the initiatives and those leading clinical services.

Our findings indicated that for individuals driving initiatives which stemmed from their personal ambition to develop research activities within their organisation, a degree of tenacity and good fortune was required, with less buy‐in from senior management at the outset. Funding, permanent role creation, high‐level endorsement, and granted protected time were reported as being key to the success of half the initiatives in this study; however, miscommunication, lack of time and permissions, competing demands, and importantly short‐term funding placed others at risk. Peckham et al. (2023) indicated the need for a systems‐based approach to create research environments that enable research involvement in health care organisations. Research drivers such as policy documents and national and local research strategies were noted in this study as important, and participants highlighted how their own research initiatives contributed to organisational research agendas and provided leverage for them to be able to operationalise these initiatives. This is reinforced by Palmer et al. (2023) who highlighted how utilising a research capacity and culture tool produced rich information to aid the development of research strategies within organisations.

Within the *initiation* phase of building research roles, there is a need to build research knowledge and confidence in nurses and midwives working in out of hospital settings. This might involve research training and development opportunities, raising awareness and engagement in research through mentorship sessions, buddying, and one‐to‐one sessions with research supervisors to share and discuss research‐related information. Multi‐faceted approaches which enable a range of activities to support clinical staff to engage in research can make a difference (Shepherd et al. 2022). The current lack of community‐based research studies does not accurately reflect the need for more research to be undertaken in this area (Bowers 2018), yet makes it difficult for nurses and midwives working in out of hospital settings to build their knowledge, confidence and skills.

The theme of linkage and collaborations aligned to the *continued growth phase* in relation to building research‐related roles in community settings. Research linkages and collaborations occurred most commonly in‐house and across organisations and mainly for cross working and learning purposes, and for study delivery and capacity building. As *continued growth* occurs, external links and networks are increasingly likely to develop. Establishing links across other networks and systems, such as academic institutions, regional research delivery networks, other practice areas, and with researchers are important and demonstrate the progression of research‐related initiatives into the growth phase of the process. Our findings highlighted that the *ambition* phase was linked to nurses and midwives taking ownership of their research contributions and raising the profile of their work through actionable dissemination. Actionable dissemination might take the form of update bulletins, training sessions, showcase events, or email distributions lists and can reach people in organisations that might not otherwise be aware of research activities and how to engage with them. Measuring the usefulness and impact of these dissemination processes is one way of evaluating their value; however, inconsistencies remain in terms of how robustly dissemination activities are evaluated (Lewis et al. 2023).

Our study identified the cross‐cutting theme of ‘trust and relationships’ which spanned the seven themes identified through the RCDi framework (Cooke 2021). Some participants spoke of how individuals were more likely to step into research if they were supported by colleagues who they trusted and felt safe with. This finding is key as it demonstrates the importance of the research environment in facilitating nurses and midwives' entry and progression through the research process. (Whitehouse et al. 2022) recognised how a number of differing organisational approaches can increase nurse involvement with research and that shared values are essential. This study found that measures to provide increased support for those new to research, such as peer support, mentoring and coaching sessions can all be useful for creating a more welcoming and inclusive research environment, as well as working to raise awareness of different research opportunities available to all nurses and midwives, regardless of their career stage or level of research experience. By investing time and resources in this, a cohort of nurses and midwives with the skills, knowledge and confidence to support, deliver and lead research in out of hospital settings is more likely to emerge, grow and be sustained.

### 5.1 Strengths and Limitations

Having a specific focus on nurses and midwives based or working in community settings was a key strength of the study and it was important that specific attention was paid to identifying this cohort of professional staff from the outset. The expert RISE steering group was pivotal in ensuring that the inclusivity of the language used within the survey was pitched at the right level, and they were well positioned to achieve the necessary reach across a variety of community settings. They were also valuable contributors in terms of critiquing the study protocol and providing feedback throughout the study process.

This study viewed nurses and midwives within community, public health, primary care and social care under the umbrella term ‘community settings’. This could be viewed as a limitation due to the wide‐ranging professional roles that are encompassed under this heading. However, similar issues relating to creating research‐related roles across these professions and settings were identified during the research process. Despite this, greater emphasis on nurses and midwives working in social care settings is required, as very few initiatives and research activities were identified in this area.

### 5.2 Implications for Practice

Viewing the RCDi framework (Cooke 2021) in a temporal arrangement lends itself well in planning change and enabling research‐related roles to be created for, and by, nurses and midwives in community settings. The evidence from this study recommends all seven RCDi principles should be applied for successful research‐related roles to be developed. In addition, more emphasis needs to be placed on building trust and relationships to enhance research environments and support people to take their first steps into research. For organisations embarking on creating research‐related roles for nurses and midwives in community settings, a ‘planning change and features of success’ template such as the one outlined in Table 4, could be used to help guide the process of research capacity building in community settings.

**TABLE 4:** Planning change questions and features for success.

Phase	Planning change questions
Initiation	Where is the research sustainability and leadership coming from? What infrastructures are in place to support the initiative? How are research skills and confidence being built?
Continued Growth	How are linkages and collaborations fostered and what do they look like?
Ambition	What are the methods to support nurses and midwives to recognise and enable, ownership and responsibility around research contributions? What does actionable dissemination look like and how will it make a difference? How can co‐production play a part in creating research‐related roles?
Throughout	Has the importance of trust and relationship building been recognised and enabled?

Phase	Features for success examples
Initiation	Collaborative working led by research leadership (R&D) alongside clinical services leadership Senior leadership acknowledgment and recognition of research drivers, strategy and policy documents. Awareness raising, building confidence and ‘tackling barriers’ through sharing and learning opportunities. Research engagement through mentoring, one‐to‐ones, workshops, buddying schemes. Building research skills and development opportunities, with a specific community focus, to enable individual progression. Funding to create roles and initiatives, protect time, enable learning and undertake opportunities. Availability of relevant and relatable community research studies. Sustainability plans for long‐term outputs to prevent initiatives being at risk.
Continued Growth	Initial focus in‐house and across organisations for cross working, learning purposes, study delivery and capacity building. Long‐term planning to establish links across networks and systems, into academic institutions, into other practice areas, with the research delivery networks.
Ambition	Recognition from an organisational perspective of research career pathways and routes into research. Enabling an environment where nurse and midwife research ownership and responsibility can develop. Profile raising employing a variety of methods from email distributions to showcasing events. Recognition of measurable outputs such as numbers of places on courses, awards received, presentations undertaken.

## 6 Conclusion

Recent years have seen a significant increase in the national drive to expand research capability and capacity in out of hospital settings (NIHR 2021), broadening the scope and opportunity for nurses and midwives to support, lead, and deliver research at all stages of their careers (NHS England and NHS Improvement 2021; NHS England 2023). Most care delivery takes place in home and community settings, with nurses and midwives best placed to incorporate research into all aspects of their day‐to‐day work (Hoverd et al. 2023). This study has identified the key enabling features of successful research‐related roles that should be considered when employers are planning research activity in community settings. This can help ensure successful new projects are developed that enable populations currently excluded from research to take part, whilst under the care of trusted and familiar health professionals.

## Author Contributions

L.W., C.H., M.A., R.E., D.R.: Made substantial contributions to conception and design, or acquisition of data, or analysis and interpretation of data; L.W., C.H., M.A., R.E.: Involved in drafting the manuscript or revising it critically for important intellectual content; L.W., C.H., M.A., R.E., D.R.: Given final approval of the version to be published. Each author should have participated sufficiently in the work to take public responsibility for appropriate portions of the content; L.W., C.H., M.A.: Agreed to be accountable for all aspects of the work in ensuring that questions related to the accuracy or integrity of any part of the work are appropriately investigated and resolved.

## Conflicts of Interest

The authors declare no conflicts of interest.

## Supporting information


Appendix S1.

Appendix S2.

Appendix S3.

Appendix S4.

Appendix S5.

Appendix S6.

## Data Availability

The data that support the findings of this study are available from the corresponding author upon reasonable request.
