# Supplementary material for: Key Features of Successful Research‐Related Roles for Nurses and Midwives in out of Hospital Settings: A Mixed Methods Approach
Source: J Adv Nurs. 2025 Jul 1;82(4):3702–15. doi: 10.1111/jan.70021 (PMC12994640; doi:10.1111/jan.70021)
Supplement: Supplementary file 1 — Appendix S1. [file JAN-82-3702-s002.pdf]

Appendix _1 _SuppInfo.pdf

RISE Survey

RISE (Research In Community Settings) - is a NIHR led project looking to engage nurses and midwives based in community, public health, primary and social care settings, who are involved in examples of good practice research initiatives and activities.

We want to identify and learn more about nursing and midwifery research roles and/or initiatives/activities already happening in community settings. These could be many and varied e.g. a district nurse undertaking a research capacity building role, a research midwife having a focus on delivering community-based research, a 0-19 Service and mental health organisation supporting research champions, or clinical academic role development for Advanced Clinical Practitioners etc.

Do you have an example of an initiative/activity in your community, public health, primary or social care setting, that has a focus on successful research engagement for nurses and midwives? If so, we would very much appreciate you completing this survey. A greater understanding of some of the initiatives/activities will help us to shape the next phase of the project, which is to undertake pilot work based on our learnings.

Your information is really valuable to us - please complete the survey by 23rd January 2023. It should take a minimum of 20 minutes.

Privacy Statement:

The NIHR is managed by a number of Coordinating Centres contracted to the DHSC. Its purpose is to fund health and care research and translate discoveries into practical products, treatments, devices and procedures, involving patients and the public in all its work. For further information please see The NIHR Website - www.nihr.ac.uk. The full NIHR Privacy Notice is available here: www.nihr.ac.uk/documents/nihr-privacy-policy/12242

Personal information provided will be held and used in compliance with the General Data Protection Regulation (GDPR) and the Data Protection Act 2018. The Department of Health and Social Care (DHSC) is the Data Controller for the NIHR under the GDPR and the Data Protection Act 2018. Under the GDPR and the Data Protection Act 2018, we have a legal duty to protect any information we collect from you. The personal information that you provide will only be shared with the RISE Project Steering Group members and NIHR Nursing and Midwifery Team, who are involved for the specific purpose outlined above. You should be aware that information given to us might be shared across the NIHR for statistical analysis and management purposes. The NIHR is committed to protecting privacy and to processing all personal information in a manner that meets the requirements of the GDPR and the Data Protection Act 2018. We will not pass your details to any third party or government department unless you give us permission to do so.

By completing the survey, you are giving NIHR permission to hold your data for the purposes of this work and by clicking on the "submit" button at the end of the questionnaire you are giving us consent to use the information that you are providing.

If, after submitting, you want to view your data and/or update it or wish it to be removed from our system you can make a request at any time to Lucy Ainsworth @ nursingandmidwifery@nihr.ac.uk

1. Email: short answer text
2. Name: short answer text
3. Employing organisation (in full): short answer text
4. Region: drop down
5. Your role: (short answer text)
6. Do you work in a community setting? Yes/no/hybrid.
7. Title/name of the initiative/role/programme: (short answer text)
8. Background to the initiative/role/programme: (short answer text)
9. Purpose/vision of the initiative/role/programme: (short answer text)
10. Which professional group does your initiative/activity relate to (i.e., community mental health nurses, paediatric nurses, practice nurses or community midwives etc) or is it more generic?
11. Is the initiative/role/programme ongoing at the present time? (if no why not): (short answer text)
12. Please provide a descriptive paragraph or 4-6 key bullet points about the initiative/activity. This might include any aims and objectives, anticipated outcomes, sources of funding required, role remits, group memberships, job outline, achievements, outputs etc. Maximum 150
13. We may want to follow up on your responses to gain greater insight into your imitative/activity - please indicate whether you would be willing for further contact from us.
