# Supplementary material for: Key Features of Successful Research‐Related Roles for Nurses and Midwives in out of Hospital Settings: A Mixed Methods Approach
Source: J Adv Nurs. 2025 Jul 1;82(4):3702–15. doi: 10.1111/jan.70021 (PMC12994640; doi:10.1111/jan.70021)
Supplement: Supplementary file 2 — Appendix S2. [file JAN-82-3702-s006.pdf]

Appendix_2_SuppInfo.pdf

Participant Information Sheet

NIHR | National Institute for Health and Care Research

RISE (**R**esearch **I**n Community **Se**ttings)

RISE – a NIHR Nursing & Midwifery project to identify features of successful research-related roles for nurses and midwives in community, public health, and social care

Information sheet for participants

This document provides information about the RISE project which is being conducted by Nursing and Midwifery at the NIHR. This NIHR led project is looking to engage with nurses and midwives based in community, public health, primary care and social care settings, and who are involved in examples of good practice research initiatives and activities.

As part of the RISE project a national survey was circulated which you took the time to complete. As a survey responder we are contacting you to request your involvement in the next phase of the project.

Before you decide whether to take part, it is important for you to understand why this next phase of the project is being undertaken and what it will involve. Please take your time to read the following information carefully and please ensure that you do not require any additional permissions to share your information. If there is anything that you are unclear about, or would like to know more information, please see the contact details at the end of this document.

What is the RISE project about?

Within community, public health, primary care and social care settings, research is less visible and opportunities for research engagement, activities and roles are less evident. The evaluation report from the 70@70 Senior Nurse & Midwife Research Leader programme (2019-2022) highlighted the need for a specific focus on community, for research impact to be made in those settings. Some examples of activities and initiatives have been identified, however it is as area of practice requiring deeper inquiry. This project aims to ‘cast its net wide’ to identify, highlight and learn from areas where successful research-related roles for nurses and midwives in community, public health, and social care are happening.

The main project aim is to explore, identify and share the key features that enable successful research-related roles for nurses and midwives in community settings.

At the end of the project, we would like to share recommendations about the key features that need to be considered when organisations are looking to empower their nurses and midwives to support, lead and delivery in research, as outlined in the CNO's strategic plan for research (NHSE&I 2021), in community settings.

Why have I been asked?

As part of the RISE national survey, you completed some baseline information that indicated you are involved in an initiative/activity in your community, public health, primary or social care setting, that has a focus on successful research engagement for nurses and midwives. Your initiative/activity has been identified within the project steering group as an example of productive practice.

Productive practice definition: An initiative or activity that demonstrates a link to research, involves nurses & midwives based in or working in a community setting and provides the potential for new learning.

We would like to learn more about your productive practice example and invite you to contribute to the next phase of the project.

Do I have to take part?

No, it is up to you to decide whether or not to take part. If you decide to take part, you will be asked to sign a consent form and you can still withdraw at any time. You do not need to give a reason if you do not participate or withdraw.

What would I have to do if you agreed to take part?

Prior to the interview you will be asked to provide written consent to participate. Once consent has been received participants will be sent an interview date/time and the interview questions to enable preparation. Participants will be invited to undertake an online interview on Microsoft Teams, with the project lead, which should take no longer than one hour. The project lead will have a topic guide with interview questions based on the issues that are important to the project aim. Agreed consent will also give permission for the RISE project steering group and sponsor to use the information provided by you for the specific purpose of the project aim (as outlined above).

What happens to the data collected?

The interview will be recorded and transcribed directly from Microsoft Teams. Once the interview recording has been transcribed, recordings will be destroyed. The transcriptions will be anonymised and analysed utilising a framework analysis approach. All other forms of data (consent forms, transcriptions) will be securely stored on the password protected NIHR Nursing & Midwifery Database for 5 years, after which

point, they will be destroyed. The project has been discussed with NIHR governance to ensure that it is GDPR compliant. HRA decision tools have confirmed that the project is research, but that REC review is not required. The project has been discussed with NIHR governance and it has been agreed that the documents and information satisfies the governance procedures required within the sponsor organisation (NIHR). The project presents no major ethical dilemmas and ethical principles in research will be adhered to in relation to informed consent, integrity, confidentiality and data protection, conflicts of interest and respect for persons.

What will happen to the findings of this project?

Findings will be written up and presented to the participants of the study following completion of the analysis. The results will also be shared with the RISE steering group and project sponsor. The results will form the basis of a report/publication for wider circulation. As a participant any report/publication will share anonymous information and will not identify you or your organisation. Any resource developed and shared from the NIHR N&M Office based on project findings is outside the scope of this particular project.

What are the possible disadvantages and risks of taking part?

We do not anticipate any disadvantages to being involved in the project and consequently that the risks are minimal.

What are the possible benefits of taking part?

The findings from the project will help the NIHR and others have a better understanding of what the key features are that enable successful research-related roles for nurses and midwives in community settings. Whilst there are no immediate benefits as a participant there is the potential opportunity to be involved in a future project to develop an intervention based on the findings of this work.

Who is organising and sponsoring this project?

The project is sponsored by the Nursing and Midwifery office at the NIHR.

Contact for further information:

Project Lead: Louise Wolstenholme NIHR N&M Clinical Fellow 07977410545

louise.wolstenholme@nhs.net
Project Support: Declan Robinson NIHR N&M Programme Coordinator

declan.robinson@nihr.ac.uk
What if I want to make a complaint?

If you want to make a complaint please contact the project lead in the first instance. If you are not happy and wish to make a formal complaint please contact Lucy Ainsworth lucy.ainsworth@nihr.ac.uk Programme Officer of Nursing & Midwifery Office.

Thank you for taking the time to read this information sheet and for considering this invitation to participate in the project.
