# Supplementary material for: Key Features of Successful Research‐Related Roles for Nurses and Midwives in out of Hospital Settings: A Mixed Methods Approach
Source: J Adv Nurs. 2025 Jul 1;82(4):3702–15. doi: 10.1111/jan.70021 (PMC12994640; doi:10.1111/jan.70021)
Supplement: Supplementary file 3 — Appendix S3. [file JAN-82-3702-s005.pdf]

**Appendix_3_SuppInfo.pdf**

**Consent form**

NIHR | National Institute for Health and Care Research

# RISE (**R**esearch **I**n Community **Se**ttings)

## Consent Form

Project Title– a NIHR Nursing & Midwifery project to identify features of successful research-related roles for nurses and midwives in community, public health, and social care.

Project Lead: Louise Wolstenholme louise.wolstenholme@nhs.net

Please initial in the boxes if you agree.

| | |
|---|---|
| 1. I confirm that I have read the information sheet for the above project. I have had the opportunity to consider the information and to ask questions and have had those answered satisfactorily. | |
| 2. I understand that my participation in this project is voluntary and that I am free to withdraw at any time or to withdraw any unprocessed data previously supplied without giving any reason. | |
| 3. I agree to the recording of the interview for the purpose of the study via Microsoft Teams | |
| 4. I understand that the project documentation whether hard copies or electronic (e.g., consent form, recordings, transcripts) will be shared only within the NIHR nursing and midwifery team and the members of the RISE steering group. | |
| 5. I understand that documents will be stored on a secure database and kept for up to 5 years and will be disposed of securely if it is confirmed that they are no longer required. | |
| 6. I agree that quotations from my interview can be used anonymously in the report for the study and conference presentation. | |
| 7. I would like a summary of the findings sent to me once the study is complete. I understand that this will mean my contact details will be kept securely on file for this purpose. | |
| 8. I agree so being recontacted by the project team to see if I would be interested in taking part in follow up work related to this project. | |
| 9. I agree to take part in the above project. | |

| **Name of Participant** | **Date** | **Signature** |
|---|---|---|
| | | |
| **Name of Project Lead** | **Date** | **Signature** |
| | | |
