# Supplementary material for: Key Features of Successful Research‐Related Roles for Nurses and Midwives in out of Hospital Settings: A Mixed Methods Approach
Source: J Adv Nurs. 2025 Jul 1;82(4):3702–15. doi: 10.1111/jan.70021 (PMC12994640; doi:10.1111/jan.70021)
Supplement: Supplementary file 4 — Appendix S4. [file JAN-82-3702-s004.pdf]

Appendix_4_SuppInfo.pdf

Invite to interview.

NIHR | National Institute for Health and Care Research

Dear [ ]

Many thanks for completing the RISE (**R**esearch **I**n Community **Se**ttings) survey in January this year.

RISE (Research In Community Settings) - is a NIHR led project looking to engage nurses and midwives based in community, public health, primary care and social care settings, who are involved in examples of good practice research initiatives and activities.

We were very interested in the information that you shared with us and are hopeful that you could attend an informal interview with the project lead to discuss your initiative/activity in more detail.

Please see attached the information sheet and consent form explaining the informal interview.

If after reviewing this information you are happy to proceed, please reply to this email attaching a signed consent form. We will then contact you to arrange a suitable date and time to meet with the Project Lead. The meeting will take place over Microsoft Teams and be recorded.

Once again, thank you for your interest in this project- we really are very keen to know more about your initiative/activity.

Best wishes

Louise Wolstenholme, NIHR N&M Clinical Fellow

Declan Robinson, NIHR N&M Programme Coordinator
