# Supplementary material for: Key Features of Successful Research‐Related Roles for Nurses and Midwives in out of Hospital Settings: A Mixed Methods Approach
Source: J Adv Nurs. 2025 Jul 1;82(4):3702–15. doi: 10.1111/jan.70021 (PMC12994640; doi:10.1111/jan.70021)
Supplement: Supplementary file 5 — Appendix S5. [file JAN-82-3702-s001.pdf]

**Appendix_5_SuppInfo.pdf**

**Interview topic guide**

RISE (**R**esearch **I**n Community **Se**ttings)

Interview Guide

Please note that the term research initiative is used throughout the questions however this could also be better described on an individual basis as a research activity or research role.

1. Can you very briefly describe the research initiative you are involved in? What is its purpose and intended outcomes?

2. How did the research initiative come about? What was the catalyst that led to its inception?

These following questions are related to factors considered to be important in building research capacity. Not all the factors will matter in equal measure.

3. How much did *skills and confidence building* matter for your nurses and/or midwives in taking part in your successful research initiative in a community setting? What did this look like? (prompts: mentoring, training, peer support, shared learning, opportunity for growth, personal interest, role models etc)

4. What were the key enabling factors that contributed to the success of the research initiative in relation to *infrastructure*? (prompts: R&D support, research interest groups/forum, champion programmes, funded opportunities etc)

5. In relation to *leadership and sustainability* and as key considerations for success:

a) Who were the key persons involved and what did they enable to ensure the success of the research initiative? (prompts: permissions, protected time, funding, roles, vision, risk taking, flexibility, route to impact. empowering etc)

b) How much did ownership and responsibility factors matter? (prompts: ownership/buy in, governance, valued, contribution)

c) How is the initiative sustained? What has been put in place or needs to be for it to continue? (prompts: roles, funding source, embedded, policy, strategies)

d) Is the initiative 'at risk' – what would stop it from being 'at risk'?

6. How important were the following factors in enabling success in your research initiative?

    a) Linkages and collaborations (prompts: researchers, NHS Trusts, research depts, HEIs, CRN, professional bodies, patient & public etc)
    b) co-production (prompts: bringing right people together, close to practice research, roles for practitioners etc)
    c) Dissemination (prompts: routes, publications, conferences, evidence into practice, visibility etc)

7. a) How did the research initiative progress over time? What lessons did you learn from the research initiative?

    b) Were there any unexpected findings or outcomes? What would you do differently next time?

8. What recommendations do you have for other organisations to support research-related initiatives for nurses and midwives in community settings? Could your initiative be scaled up and/or transferable to other areas?

9. Do you have any additional comments or insights on the research initiative you were involved in?
