# Supplementary material for: Key Features of Successful Research‐Related Roles for Nurses and Midwives in out of Hospital Settings: A Mixed Methods Approach
Source: J Adv Nurs. 2025 Jul 1;82(4):3702–15. doi: 10.1111/jan.70021 (PMC12994640; doi:10.1111/jan.70021)
Supplement: Supplementary file 6 — Appendix S6. [file JAN-82-3702-s003.pdf]

Appendix_6_SuppInfo.pdf

Themes and subthemes

Themes	Skills & Confidence Building	Infrastructure	Sustainability and Leadership	Ownership & responsibilities	Linkages and Collaborations	Co-production	Actionable Dissemination
Subthemes and descriptors	Awareness raising	Studies	Organisational leadership	Initiative not owned.	Researcher links	Shared Working	Profile raising/visibility.
	Evidence of awareness raising for the individual, within a team, the organisation or across systems. Sharing of information	Availability of appropriate studies	Evidence of organisational leaders wanting to embed research, have a research culture, strategy n=7 (n=2 that felt a lack of organisational leadership	Evidence of top-down approaches , creating buy-in	Evidence of links with researchers to deliver studies	Evidence of working co-productively to achieve a shared aim	Evidence of sharing to profile raise, to create kudos, celebratory, worthwhile
	Research Engagement	Role/initiatives created.	Drivers	Ownership from initiation	R&D managers/teams	Ambition	Outputs
	Evidence of activity that is beyond raising awareness e.g., mentoring, workshops, 1:1s	Evidence of initiatives /roles that have been created to enable successful research-related activities e.g., champions, workshops, journal clubs	Policy documents, drivers influencing research-related activities	Evidence of initiative being led bottom up	Evidence of links with R&D teams	Co-production not in place, have the potential to make a difference	Evidence of outputs/impacts for the initiative/work undertaken e.g., conferences, blogs, champion programme
	Confidence building	Funding	Funding & time	Creating ownership & enabling responsibility	Organisational links	Not in place	Creating an identity
	Evidence of confidence building through sharing new skills with others, applying existing skills in new situation, working with other professional	Availability of funding required to enable infrastructure for the research activity	Funding & time required for the initiative to be sustained	Evidence of leadership support to enable ownership and responsibility	Evidence of links across the organisation/services for learning purposes, cross working	Less appetite for this at present. Not the aim of the initiative	Evidence of sharing work to help create an identity, feelings of ownership & responsibility

	Skills developed	R&D involvement	Clinical /Services role	Ownership ambition	HEI/academic links		Scalability & transferability
	Evidence of skills developed (and how)	Evidence of R&D supporting the initiative with resources	Evidence of clinical/services support mattering, protected time, permissions	Evidence of ownership and responsibility mattering but not there	Evidence of working (or ambition to) with Heis /from HEI perspective		Evidence that the initiative could be scaled &/or transferred
	Development opportunities	Virtual/digital	Distributed Leadership	Personal Growth	CRN/practice links		Ambition to disseminate.
	Evidence of progressive skill development	Evidence of use of virtual working in relation to infrastructure	Evidence of leadership 'involvement' across the different levels mattering	Evidence of personal growth, tenacity, self-directed	Evidence of working with/between the NIHR/CRN and practice (or ambition to)		Lack of availability or resources, recognises potential but limited
	Barriers	Establishing links	Leadership from research	Luck	Networks & Systems working		
	Responses to research involvement e.g., fear, reluctance, apprehension, approach from others, misconception	Right people at right level	Evidence of R&D, research leaders supporting activity	Feelings of being fortunate, not planned, right time right place	Evidence of working across systems or regions		
		Community infrastructure	Long term outcomes		Establishing links		
		Evidence of infrastructure in community services to be able to support research, geography, buildings	Evidence of sustainability mattering, impacts, potential risks		Evidence of a drive to have or create linkages with the relevant people for relevant reasons		
			Communication				
			Evidence of communication/miscommunication at/within leadership levels				
